# Association of Food Insecurity with Nutrient Intake and Depression among Korean and US Adults: Data from the 2014 Korea and the 2013–2014 US National Health and Nutrition Examination Surveys

**DOI:** 10.3390/ijerph18020506

**Published:** 2021-01-09

**Authors:** Seung Jae Lee, Kyung Won Lee, Mi Sook Cho

**Affiliations:** 1 Department of Nutritional Science and Food Management, Ewha Womans University, Seoul 03760, Korea; seungjae11@hanmail.net; 2 Department of Home Economics Education, Korea National University of Education, Cheongju 28173, Korea; kwlee@knue.ac.kr

**Keywords:** food insecurity, nutrient intake, depression, adult, KNHANES, NHANES

## Abstract

The purpose of this study is to understand the current status of food insecurity in Korea and the US and to compare the relationship of food insecurity with nutrient intake and depression. Data from the 2014 Korea and the 2013–2014 US National Health and Nutrition Examination Surveys were analyzed, and a total of 3102 Korean and 3234 American adults aged 20–64 years were included. Study subjects were classified into three groups according to degree of food insecurity assessed by the 18-item Household Food Security Survey Module: food secure (FS), mildly food insecure (FI 1), and moderately-to-severely food insecure (FI 2) groups. Energy and nutrient intake were assessed using a 24-h dietary recall. Depression was measured using the Patient Health Questionnaire-9 (PHQ-9) screener. The prevalence of food insecurity was 17.2% in Korea and 26.4% in the US. In both countries, the people in the FI 2 group had lower incomes and education levels and were mostly single. Energy and nutrient intake differed by food insecurity status. In both Korea and the US, adults with moderate-to-severe food insecurity (FI 2) consumed fewer proteins, fiber, potassium, and vitamin C. Additionally, the FI 2 groups had higher proportions of people not meeting the Dietary Reference Intake for protein, potassium, niacin, and vitamin C than the FS groups in Korea and the US. FI 2 people were three times more likely to be depressed than FS group; this difference was stronger in Korea than the US. We found that the prevalence of food insecurity was higher in the US than in Korea, and food insecurity was associated with reduced nutrient intake and increased odds of depression in both Korean and US adults. Therefore, food insecurity is an important public health issue at both the individual and national levels. Continuous monitoring and new intervention activities to promote food security are needed.

## 1. Introduction

The term “food insecurity” was introduced by Campbell in 1990 [1] to indicate a limited availability of nutritionally adequate and safe foods or a limitation or uncertainty in the ability to acquire acceptable foods in socially acceptable ways [2,3]. Food insecurity is one of the most significant global public health issues, and poor nutrition is a critical cause of chronic health issues, including mental and physical health problems [4,5,6].

Despite rapid economic development, food insecurity prevalence was 14.3% among Americans in 2013 [7]. In Korea, a recent study using data from the fifth Korea National Health and Nutrition Examination Survey (KNHANES) reported that food insecurity prevalence was 11.3% among Korean adults [8]. Another study of homeless families from France showed that only 14.0% were food secure, 43.3% had low food security, and 9.8% had very low food security [9]. In particular, it has been reported that food insecurity is more serious in low-income countries. In South Africa [10] and rural Ethiopia [11], the prevalences of household food insecurity were 38% and 33%, respectively. In addition, in India, nearly half of households suffered food insecurity, and 40% and 4.5% of households were moderately and severely food insecure, respectively [12].

The negative effects of food insecurity across the life course are a serious, yet preventable, public health issue [13]. Food insecurity is associated with poor nutritional [14,15], physical [16,17,18], and psychosocial [19,20,21] wellbeing [22] for children, adults, families, and communities. Many studies have concluded that food insecurity is associated with unfavorable health outcomes [8,23,24,25,26,27,28] and can cause malnutrition due to inadequate nutrient intake [8,23]. Additionally, depression is one of the most common mental health problems in the world, affecting more than 4.4% of the global population [29]. Several studies have indicated that food insecurity is correlated with mental health problems such as mood disorders and depressive symptoms [4,20,30,31,32,33].

Therefore, this study aimed to measure food insecurity status and investigate the relationships of food insecurity with nutrient intake and depression in Korean and American adults aged 20–64 years using the 2014 KNHANES and 2013–2014 US National Health and Nutrition Examination Survey (NHANES) data.

## 2. Materials and Methods

### 2.1. Data Sources and Study Populations

The Korea and US National Health and Nutrition Examination Surveys (KNHANES and NHANES, respectively) were conducted to evaluate the health and nutritional status of adults and children in each country. The KNHANES and the NHANES consists of health examination, health interview, and dietary data. The surveys were conducted by each country’s Centers for Disease Control and Prevention (CDC) and data are publicly available.

The 2014 Korea and 2013–2014 US NHANES data were analyzed in the current study. A total of 4311 Korean adults aged 20–64 years from 2014 KNHANES were included. We excluded participants who reported implausible energy intakes (<500 kcal/day or ≥5000 kcal/day; *n* = 581), who were pregnant (*n* = 130), or who had missing covariate data (*n* = 529). For the US, 8758 participants aged 20–64 years from the 2013–2014 NHANES were included. We excluded participants for the same reasons given above: individuals who listed <500 kcal or ≥5000 kcal per day (*n* = 2156), who were pregnant (*n* = 78), or were missing covariates (*n* = 3290). After all exclusions, 3102 Korean adults (men: 1201, women: 1901) and 3234 US adults (men: 1595, women: 1639) were included in the final analyses.

The KNHANES was approved by the institutional review board of the Korea CDC (2013-12EXP-03-5C) and the NHANES was approved by CDC/NCHS Ethics Review Board (Continuation of Protocol #2011-17). All participants gave informed consent.

### 2.2. Food Insecurity

Since 1999, the US NHANES has assessed household food security through the Household Food Security/Hunger Survey Module (HFSSM). Household food insecurity was measured from one questionnaire in the KNHANES until 2011. However, since 2012, the 18-item questionnaire that was used in the US HFSSM has begun to be used in the KNHANES. Therefore, in this study, data based on the 18-item HFSSM were used for both Korea and the United States analyses.

The questionnaire for assessing food insecurity consists of 10 items including three household-referenced questions and seven adult-referenced questions. For households with children, eight additional child-referenced questions were surveyed. Total score distributions range from 0 to18 for households with children, and from 0 to 10 for households without children to measure food insecurity. Food insecurity status was classified into three groups in both countries based on the sum of the scores used in previous studies [4,34]. For both the Korean and US participants, a score of 0–2 for both households with/without children was classified as the food secure (FS) group, a score of 3–7 for households with children and a score of 3–5 for households without children were classified as the food insecure 1 (FI 1; mildly food insecure) group, and a score of 8–18 for households with children and a score of 6–10 for households without children were classified as the food insecure 2 (FI 2; moderately-to-severely food insecure) group.

### 2.3. Nutrient Intake Assessment

Both the KNHANES and NHANES have 24-h dietary recall interviews for participants of all ages. To understand the intake of energy and nutrients according to food insecurity status, we used single 24-h dietary recall data. The percentage of energy intake from carbohydrates, protein, and fat was evaluated to investigate their contribution to total daily energy intake. Daily nutrient intakes, including total energy intake, carbohydrates, protein, fat, dietary fiber, calcium, phosphorus, iron, sodium, potassium, vitamin A, vitamin B1, vitamin B2, niacin, and vitamin C, were calculated. Additionally, the proportions of participants with nutrient intakes below and above the Dietary Reference Intakes (DRIs) in both Korean and US adults were estimated. Estimated average requirements (EARs) for protein, calcium, phosphorus, iron, vitamin A, vitamin B1, vitamin B2, niacin, and vitamin C were compared according to gender- and age-specific nutritional intake standards. Energy intake was compared using the estimated energy requirement (EER), and the adequate intake (AI) was used for dietary fiber, sodium, and potassium.

### 2.4. Definition of Depression

Depression was measured using the Patient Health Questionnaire-9 (PHQ-9), developed by Spitzer et al. [35]. The PHQ-9 is a self-report instrument to measure the frequency of depression symptoms in the past two weeks. The PHQ-9 contains nine items, with each item being determined on a four-point scale ranging from 0–3 (0 = “not at all,” 1 = “several days,” 2 = “more than half the days,” 3 = “nearly every day”). The score distribution ranges from 0–27, and high score means severe depression. Based on a threshold score of 10, participants with a PHQ-9 score ≥ 10 were defined as “depressed,” and those having a PHQ-9 score < 10 were classified as “not depressed.”

### 2.5. Potential Covariates

The following covariates were entered into the analytic models for Korean and US adults in this study: gender (men and women), age (20–29, 30–49, and 50–64 years), income level (low, medium, and high), education level (middle school or less, high school, and college or higher), marital status (married and single including separated, divorced, widowed, and stayed unmarried), race/ethnicity (only for NHANES; non-Hispanic white, non-Hispanic black, Hispanic, and others including Asian, American Indian or Alaska Native, Native Hawaiian or other Pacific Islander), weight status (calculated as weight (kg) divided by squared height (m) (kg/m^2^); underweight < 18.5 kg/m^2^, normal weight 18.5–23 kg/m^2^, overweight 23–25 kg/m^2^, and obese ≥ 25 kg/m^2^ for the Korean participants based on the criteria for the Asia-Pacific region of the World Health Organization [36], underweight < 18.5 kg/m^2^, normal weight 18.5–25 kg/m^2^, overweight 25–30 kg/m^2^, and obese ≥ 30 kg/m^2^) for the US participants based on the criteria from the Centers for Disease Control and Prevention [37], the presence of chronic diseases (yes or no; individuals having type 2 diabetes, hypertension, or metabolic syndrome were classified as having chronic diseases), physical disabilities (yes or no), and menopausal status (only for women; yes or no).

### 2.6. Statistical Analyses

The SAS 9.4 version statistics package (Statistical Analysis System, SAS Institute Inc., Cary, NC, USA) was used for analyses. The Korea and US NHANES data had a multi-stage stratified probability sampling design. To account for the complex sampling design for both national surveys, PROC SURVEY procedures including strata, clusters, and weights were applied. For this study, *p*-values < 0.05 were considered statistically significant.

Descriptive statistics, including means, frequencies, and proportions, were calculated, and a SURVEYFREQ procedure was conducted to examine the association between socio-demographic characteristics and the proportions of nutrient intake not meeting the DRIs according to food insecurity status. The SURVEYREG procedure was used to compare differences in the percentage of energy intake from carbohydrates, protein, and fat and in the daily actual nutrient intake adjusted for chronic disease, physical disabilities, and menopausal status (only in women) according to food insecurity status. Using the SURVEYLOGISTIC procedure, multiple logistic regression analysis was conducted to estimate odds ratios (ORs) and 95% confidence intervals (CIs) for depression adjusted for confounders (model 1: no adjustment; model 2: sex and age in both countries; model 3: sex, age, income, marital status, education level, chronic disease, physical disabilities, and menopausal status (only in women) in KNHANES, and sex, age, income, marital status, education level, race/ethnicity, chronic disease, physical disabilities, and menopausal status (only in women) in NHANES).

## 3. Results

**Figure 1:**
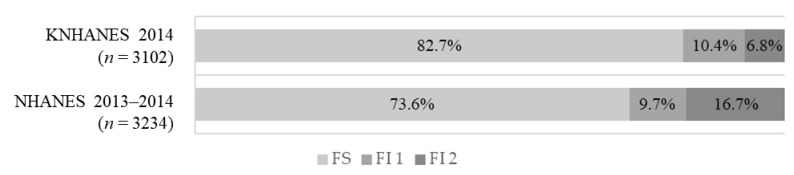
Prevalence of food insecurity in the 2014 Korea National Health and Nutrition Examination Survey (KNHANES) and 2013–2014 US National Health and Nutrition Examination Survey (NHANES) according to food insecurity status. The study subjects were divided into three groups according to their food insecurity status: (1) food secure group (FS), (2) mildly food insecure group (FI 1), (3) moderately-to-severely food insecure group (FI 2).

Food insecurity status among the Korean and US adults aged 20–64 years is shown in Figure 1. Among the 3102 Korean participants, 17.2% (*n* = 527) were food insecure (10.4% for FI 1, 6.8% for FI 2). Among the 3234 American participants, 26.4% (*n* = 1062) were in the food insecure group (9.7% for FI 1, 16.7% for FI 2).

The socio-demographic characteristics of the study populations according to food insecurity status are shown in Table 1. In Korean adults, income level, marital status, and educational level were significantly associated with food insecurity. Similarly, age, income level, marital status, education level, and race/ethnicity were associated with food insecurity status among US adults.

Energy percentages from carbohydrates, protein, and fat and nutrient intake by food insecurity status are presented in Table 2. In Korea, there was no differences in the proportion of energy from carbohydrates, protein, and fat among food insecurity groups, whereas among Americans, the FI 2 group consumed a significantly higher percentage of energy from carbohydrates (50.9%) and a lower percentage from protein (15.3%) and fat (33.8%) compared with the FS group (carbohydrates:protein:fat = 48.2%:16.6%:35.2%). Daily nutrient intake significantly differed by food insecurity status in both Korean and US adults. In Korean adults, people in the FI 2 group were less likely to consume energy, carbohydrates, protein, fiber, calcium, phosphorus, iron, sodium, potassium, and vitamin C compared with those in the FS group. In the US, people experiencing food insecurity tended to consume less protein, fiber, potassium, vitamin A, and vitamin C than food secure people. However, regarding carbohydrates, the food insecure groups consumed more than the FS group. Overall, the moderately-to-severely food insecure group (FI 2) had significantly lower consumption of protein, fiber, potassium, vitamin A, and vitamin C than the other groups.

**Figure 2:**
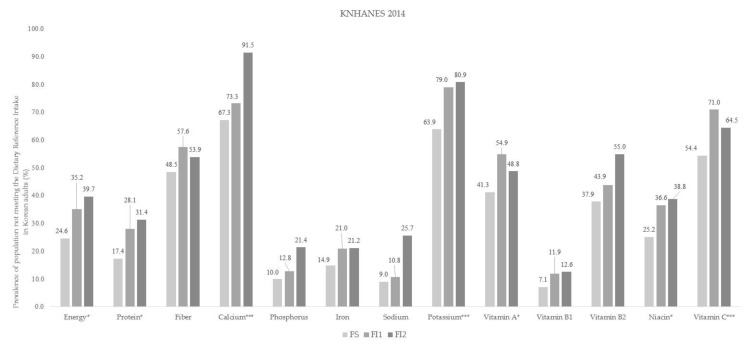
Prevalence of population not meeting the Dietary Reference Intake according to food insecurity status in the Korean adults. The study subjects were divided into three groups according to their food insecurity status: (1) food secure group (FS), (2) mildly food insecure group (FI 1), (3) moderately-to-severely food insecure group (FI 2); *p*-value was obtained from the Chi-square test (* *p* < 0.05, *** *p* < 0.001); KNHANES: Korea National Health and Nutrition Examination Survey.

**Figure 3:**
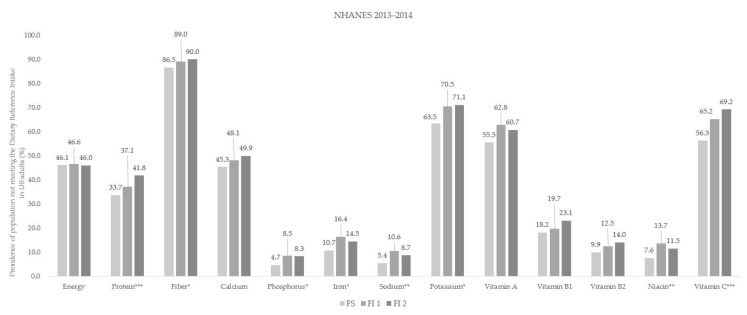
Prevalence of population not meeting the Dietary Reference Intake according to food insecurity status in the US adults. The study subjects were divided into three groups according to the food insecurity status: (1) food secure group (FS), (2) mildly food insecure group (FI 1), (3) moderately-to-severely food insecure group (FI 2); *p*-value was obtained from the Chi-square test (* *p* < 0.05, ** *p* < 0.01, *** *p* < 0.001); NHANES: National Health and Nutrition Examination Survey.

The prevalence of people not meeting the DRIs based on daily energy and nutrient intake according to food insecurity status in both countries is shown in Figure 2 and Figure 3. Among Korean adults, the FI 2 group had the largest proportion of people not meeting the DRIs for energy, protein, calcium, potassium, vitamin A, niacin, and vitamin C compared with the FS group (Figure 2). In the US, the percentage of participants not meeting the DRIs for protein, fiber, phosphorus, iron, sodium, potassium, niacin, and vitamin C was higher in both the FI 1 group and FI 2 group than in the FS group (Figure 3).

The significant associations between food insecurity status and depression are shown in Table 3. After adjusting for sex, age, race/ethnicity (only for US adults), income, marital status, education level, the presence of chronic diseases, physical disabilities, and menopausal status (only for women) (model 3), Korean adults in the FI 1 group and the FI 2 group had 1.9 times (95% CI: 1.1–3.3) and 4.0 times (95% CI: 1.7–9.0) higher odds of depression, respectively, than people in the FS group (*p* < 0.0001). Likewise, the US adults in the food insecurity groups had significantly higher odds of depression (FI 1: OR 2.0, 95% CI 1.1–3.6; FI 2: OR 3.0, 95% CI 2.3–3.7; *p* < 0.0001).

## 4. Discussion

This study identified and compared the current status of food insecurity in Korean and American adults aged 20–64 years using data from the 2014 KNHANES and the 2013–2014 NHANES. The association of food insecurity with nutritional intake and depression was separately analyzed in both countries. Food insecurity was investigated using the 18-item HFSSM. The prevalence of Korean and American adults with food insecurity (FI groups) was 17.2% and 26.4%, respectively. These findings were consistent with previous studies of Korean [8], American [38], and Canadian adults [39]. In particular, the FI 2 group, which has moderate-to-severe food insecurity, occurred at higher rates among Americans (16.7%) compared with Koreans (6.8%). Income level, marital status, and educational level were significantly associated with food insecurity in Korean and American adults. Compared with the FS group, the FI 2 group tended to be single and have lower incomes and education levels, and low-income level was strongly associated with food insecurity in this study. Previous studies also found economic status to be a predictor of food insecurity. More severe food insecurity has consistently been reported to be associated with lower household income [40,41] and socioeconomic status [42]. Frequency of food insecurity was higher in socio-economically disadvantaged groups [43,44,45].

Energy intake from carbohydrates, protein, and fat by food insecurity status was compared, and total energy intake and the %energy from carbohydrates were higher in the FI 2 group compared with the FS group in the US (FI 2: 50.9%, FI 1: 49.6%, FS: 48.2%), but no significant differences were found in Korea. It can be explained by the higher intake of low-cost, high-calorie foods low-income and food insecure groups in the US [46]. Additionally, different associations might be due to differences in the recommended %energy from carbohydrates in the two countries (55–65% for Korean adults; 45–65% for US adults) [47,48]. In the FI 2 group, carbohydrates accounted for 63.7% of total energy intake in Korean adults but it was 50.9% in American adults. These patterns were observed in other groups.

Food insecure group consumed most nutrients less than recommended levels in both Korea and the US, indicating that malnutrition due to food insecurity was a serious problem. In this study, food insecure groups had lower nutrient intake and, in both Korean and American adults, intakes of protein, fiber, potassium, and vitamin C were significantly lower in the FI 2 group than the FS group. Based on the DRIs, the proportion of food insecure groups who consumed inadequate nutrients was usually higher in both countries for protein, potassium, niacin, and vitamin C. Previous studies also found that people with food insecurity showed inadequate intake of vitamins A, E, and C, folate, and magnesium [49]. Given that adequate nutrient intake is essential to maintaining health, food insecurity, which can lead to extreme hunger beyond undernutrition or malnutrition, can become a fundamental survival problem.

We also found that food insecurity was strongly related to depression according to participants’ responses to the PHQ-9 questionnaire. In the 2014 KNHANES data, the PHQ-9 questionnaire was firstly introduced to diagnose depression, which improved the reliability of depression diagnoses compared with previous years that relied on a self-written one-question response [50]. After adjustment for various confounding factors, significant associations between food insecurity and depression remained. In both countries, the odds of depression increased by more than 300% in the FI 2 group compared with the FS group. This was in line with other studies that reported a strong association with food insecurity and depression [51,52]. One study further concluded that food insecurity not only causes depressed mood, but also maintains depression [53]. The current literature mentions that food insecurity results in, or is associated with‚ poor mental health outcomes, including depression [20,31,34,54]. Thus, the relationship between food insecurity and poor health conditions, especially insufficient nutritional status or depression, has been shown to have a bi-directional effect: depression can cause food insecurity, which can act as a factor that worsens depression. Inadequate nutrition and depression have the same relationships as well [52].

This study has some limitations. First, causal relationships among food insecurity, depression, and nutrient intake cannot be determined from the cross-sectional design of the Korea and US NHANES. Second, it was difficult to directly compare the associations of food insecurity with depression between Korea and the US. Although two main variables, food insecurity (exposure variable) and depression (outcome variable), were assessed using the same questionnaires, there were differences in the various covariates, such as household income level and weight status, included in the analytical models. Moreover, cultural and environmental differences between the two countries also make it difficult to compare these associations. Thus, it may be erroneous to simply compare and explain the results of the data without taking this into account, and our findings should be interpreted cautiously. Finally, this study only investigated whether people were depressed, not their degree of depression. A detailed follow-up analysis of food insecurity and depression that explores differences by gender or age would be useful. However, despite these limitations, using the highly credible and nationally representative samples from Korea and the US, we showed that food insecurity was prevalent in both Korean and American adults. Furthermore, this study investigated the relationship of not only food insecurity, but also nutritional intake, with depression, and nutrient intakes were estimated to identify inadequate nutrient intakes across food insecurity status compared with the DRIs in both countries.

## 5. Conclusions

In conclusion, prevalence of food insecurity was found to be higher in the US than in Korea among adults aged 20–64 years. Food insecurity was associated with poorer nutrient intake and higher odds of depression in both countries. Our findings provide insight into intervention programs and policies to ensure food security. Recognizing that food insecurity is still an important public health concern, continuous monitoring of food insecurity status in both countries is needed.

## Figures and Tables

**Table 1:** Socio-demographic characteristics of the study population by food insecurity status in the Korean and US adults.

	KNHANES 2014				*p*-Value ^2^	NHANES 2013–2014				*p*-Value
	FS ^1^ (*n* = 2575)	FI 1	FI 2	Total		FS	FI 1	FI 2	Total	
		(*n* = 315)	(*n* = 212)	(*n* = 3102)		(*n* = 2172)	(*n* = 366)	(*n* = 696)	(*n* = 3234)	
Sex										
Men	1007 (49.7) ^3^	123 (47.8)	71 (45.1)	1201 (49.9)	0.3563	1083 (50.7)	176 (49.7)	336 (48.8)	1595 (50.3)	0.8178
Women	1568 (50.3)	192 (52.2)	141 (54.9)	1901 (50.1)		1089 (49.3)	190 (50.3)	360 (51.2)	1639 (49.7)	
Age										
20–29	344 (20.4)	46 (25.4)	29 (21.8)	419 (21.0)	0.2841	424 (21.0)	93 (28.0)	171 (30.0)	688 (23.2)	0.0014
30–49	1251 (49.6)	139 (43.5)	84 (41.7)	1474 (48.4)		951 (42.1)	169 (47.8)	322 (44.8)	1442 (43.1)	
50–64	980 (30.0)	130 (31.2)	99 (36.6)	1209 (30.6)		797 (37.0)	104 (24.2)	203 (25.2)	1104 (33.8)	
Income Level										
Low	488 (19.5)	113 (37.3)	140 (66.3)	741 (24.6)	<0.0001	752 (25.8)	249 (62.2)	563 (74.4)	1564 (37.4)	<0.0001
Medium	630 (24.7)	105 (31.2)	51 (22.6)	786 (25.2)		484 (22.1)	86 (28.2)	107 (19.4)	677 (22.2)	
High	1457 (55.8)	97 (31.5)	21 (11.1)	1575 (50.2)		936 (52.2)	31 (9.6)	26 (6.2)	993 (40.4)	
Marital Status ^4^										
Married	1984 (71.2)	221 (65.1)	115 (51.2)	2320 (69.2)	0.0011	1234 (59.9)	170 (48.3)	261 (38.6)	1665 (55.2)	0.0006
Single	591 (28.8)	94 (34.9)	97 (48.8)	782 (30.8)		938 (40.1)	196 (51.7)	435 (61.4)	1569 (44.8)	
Education Level										
Middle school or less	435 (12.2)	95 (21.9)	73 (27.0)	603 (14.2)	<0.0001	299 (9.9)	92 (20.2)	215 (27.2)	606 (13.8)	<0.0001
High school	1379 (56.6)	166 (61.5)	111 (59.3)	1656 (57.3)		424 (19.3)	100 (29.3)	188 (27.2)	712 (21.5)	
College or higher	761 (31.2)	54 (16.6)	28 (13.7)	843 (28.5)		1449 (70.9)	174 (50.5)	293 (45.6)	1916 (64.7)	
BMI ^5^										
Underweight	125 (4.3)	14 (5.8)	3 (7.5)	142 (4.4)	0.7084	36 (1.3)	6 (2.3)	20 (2.9)	62 (1.6)	0.3323
Normal weight	1235 (42.2)	64 (41.0)	13 (32.5)	1312 (42.0)		639 (28.5)	98 (25.9)	170 (24.9)	907 (27.7)	
Overweight	651 (22.1)	41 (20.1)	11 (31.5)	703 (22.1)		705 (33.4)	112 (31.5)	194 (27.0)	1011 (32.1)	
Obese	879 (31.4)	53 (33.2)	13 (28.4)	945 (31.5)		792 (36.9)	150 (40.3)	312 (45.3)	1254 (38.6)	
Race/Ethnicity ^6^										
Non-Hispanic white						951 (69.7)	130 (50.7)	278 (51.7)	1359 (64.9)	<0.0001
Non-Hispanic black						415 (9.4)	81 (15.2)	161 (16.6)	657 (11.2)	
Hispanic						450 (12.8)	111 (26.0)	211 (25.6)	772 (16.2)	
Other						356 (8.0)	44 (8.0)	46 (6.1)	446 (7.7)	

^1^ The study subjects were divided into three groups according to the food insecurity status: (1) food secure group (FS), (2) mildly food insecure group (FI 1), (3) moderately-to-severely food insecure group (FI 2) ^2^ *p*-value was obtained from the Chi-square test ^3^ Unweighted sample size (weighted percentage) ^4^ Marital status was divided into two groups: married and single (including separated, divorced, widowed, and stayed unmarried) ^5^ Body mass index (BMI) as weight (kg) divided by squared height (m) (kg/m^2^); by the Asia-Pacific region of the World Health Organization (WHO) as underweight: <18.5 kg/m^2^; normal weight: 18.5 to <23 kg/m^2^; overweight: 23 to <25 kg/m^2^; and obese: ≥25 kg/m^2^ in Korea and by the CDC as underweight: <18.5 kg/m^2^; normal weight: 18.5 to <25 kg/m^2^; overweight: 25 to <30 kg/m^2^; and obese: ≥30 kg/m^2^ in the US. ^6^ Other: Asian, American Indian or Alaska Native, Native Hawaiian or other Pacific Islander. KNHANES: Korea National Health and Nutrition Examination Survey; NHANES: National Health and Nutrition Examination Survey; BMI: body mass index.

**Table 2:** Energy and nutrient intake according to food insecurity status in the Korean and US adults.

	KNHANES 2014				*p*-Value ^2^	NHANES 2013–2014				*p*-Value
	FS ^1^	FI 1	FI 2	Total		FS	FI 1	FI 2	Total	
	(*n* = 2575)	(*n* = 315)	(*n* = 212)	(*n* = 3102)		(*n* = 2172)	(*n* = 366)	(*n* = 696)	(*n* = 3234)	
Energy Source Percentage										
Carbohydrate (%)	63.6 ± 0.3 ^3^	64.8 ± 1.1	62.5 ± 2.6	64.4 ± 1.0	0.4552	48.2 ± 0.4 ^a4^	49.6 ± 0.7 ^ab^	50.9 ± 0.5 ^b^	48.8 ± 0.3	0.0003
Protein (%)	14.8 ± 0.1	14.4 ± 0.3	16.0 ± 0.9	14.7 ± 0.3	0.2034	16.6 ± 0.2 ^a^	15.5 ± 0.3 ^b^	15.3 ± 0.2 ^b^	16.3 ± 0.2	0.0008
Fat (%)	21.6 ± 0.2	20.8 ± 1.0	21.5 ± 2.1	20.9 ± 0.9	0.7307	35.2 ± 0.3 ^a^	34.9 ± 0.6 ^ab^	33.8 ± 0.4 ^b^	34.9 ± 0.2	0.0168
Daily Actual Nutrient Intake										
Energy (kcal)	2157.4 ± 21.7 ^a^	1944.4 ± 78.2 ^b^	1865.3 ± 160.4 ^b^	1965.0 ± 73.71	0.0166	2150.2 ± 29.3	2184.9 ± 56.5	2210.4 ± 34.0	2163.3 ± 24.7	0.4358
Carbohydrate (g)	320.0 ± 3.1 ^a^	299.8 ± 13.6 ^ab^	260.4 ± 18.4 ^b^	295.2 ± 12.2	0.0033	252.0 ± 3.8 ^a^	259.2 ± 9.0 ^ab^	274.0 ± 4.9 ^b^	256.3 ± 3.2	0.0060
Protein (g)	75.9 ± 0.9 ^a^	67.2± 3.3 ^b^	73.1 ± 8.2 ^ab^	69.4 ± 3.2	0.0481	86.6 ± 0.8 ^a^	82.8 ± 1.2 ^b^	83.0 ± 1.8 ^ab^	85.7 ± 0.7	0.0275
Fat (g)	50.2 ± 0.8	43.6 ± 2.7	48.9 ± 10.6	45.8 ± 3.0	0.0657	83.5 ± 1.4	83.4 ± 2.3	82.6 ± 1.6	83.3 ± 1.1	0.9180
Dietary fiber (g)	25.7 ± 0.3 ^a^	21.5 ± 1.1 ^b^	21.0 ± 2.0 ^b^	21.2 ± 1.0	0.0005	17.7 ± 0.3 ^a^	16.3 ± 0.7 ^a b^	15.6 ± 0.4 ^b^	17.2 ± 0.2	0.0028
Calcium (mg)	516.1 ± 5.9 ^a^	477.4 ± 28.5 ^ab^	380.9 ± 45.0 ^b^	463.5 ± 25.1	0.0075	997.8 ± 15.9	942.0 ± 27.1	987.0 ± 36.0	990.8 ± 12.8	0.1679
Phosphorus (mg)	1159.5 ± 11.5 ^a^	1022.5 ± 43.2 ^b^	991.9 ± 73.4 ^b^	1028.3 ± 39.1	0.0029	1444.1 ± 17.1	1391.8 ± 22.8	1387.1 ± 32.7	1429.9 ± 14.3	0.1711
Iron (mg)	18.8 ± 0.8 ^a^	16.3 ± 1.2 ^ab^	14.1 ± 1.2 ^b^	16.0 ±1.1	0.0013	15.0 ± 0.2	14.4 ± 0.8	14.3 ± 0.3	14.8 ± 0.2	0.3701
Sodium (mg)	4097.3 ± 61.4 ^a^	3657.9 ± 184.5 ^b^	3254.8 ± 354.9 ^b^	3657.8 ± 171.9	0.0144	3612.3 ± 38.7	3529.6 ± 83.3	3595.5 ± 83.9	3601.9 ± 31.2	0.7098
Potassium (mg)	3298.9 ± 39.6 ^a^	2691.0 ± 123.7 ^b^	2487.4 ± 174.5 ^b^	2647.6 ± 106.9	<0.0001	2741.2 ± 34.6 ^a^	2544.9 ± 53.8 ^b^	2448.3 ± 35.6 ^b^	2675.0 ± 29.1	<0.0001
Vitamin A (μg RAE)	844.4 ± 30.0	888.8 ± 161.7	661.7 ± 115.6	853.3 ± 130.0	0.3185	655.0 ± 17.2 ^a^	568.8 ± 30.3 ^b^	579.5 ± 24.3 ^b^	634.7 ± 14.4	0.0320
Vitamin B_1_ (mg)	2.2 ± 0.0	1.9 ± 0.1	2.1 ± 0.3	2.0 ± 0.1	0.0915	1.7 ± 0.0	1.6 ± 0.0	1.6 ± 0.0	1.7 ± 0.0	0.1418
Vitamin B_2_ (mg)	1.5 ± 0.0	1.4 ± 0.1	1.4 ± 0.2	1.4 ± 0.1	0.2378	2.2 ± 0.0	2.1 ± 0.1	2.1 ± 0.1	2.2 ± 0.0	0.1448
Niacin (mg)	17.9 ± 0.2 ^a^	15.0 ± 0.6 ^b^	17.0 ± 2.4 ^ab^	15.6 ± 0.7	0.0003	27.1 ± 0.4	25.9 ± 1.1	26.8 ± 0.9	26.9 ± 0.4	0.6136
Vitamin C (mg)	115.1 ± 4.0 ^a^	68.8 ± 6.2 ^b^	64.6 ± 16.4 ^b^	64.7 ± 6.3	<0.0001	79.8 ± 1.9 ^a^	81.6 ± 4.7 ^a^	66.2 ± 2.4 ^b^	77.7 ± 1.6	0.0003

^1^ The study subjects were divided into three groups according to their food insecurity status: (1) food secure group (FS), (2) mildly food insecure group (FI 1), (3) moderately-to-severely food insecure group (FI 2) ^2^ *p*-value was obtained from the ANOVA ^3^ Mean ± standard deviation adjusted for chronic disease, physical disabilities, and menopausal status (only in women) ^4^ a,b: Means with different superscripts are significantly different by Tukey’s multiple range test. KNHANES: Korea National Health and Nutrition Examination Survey; NHANES: National Health and Nutrition Examination Survey.

**Table 3:** Association between food insecurity status and depression in the Korean and US adults.

	KNHANES 2014			*p* Value ^2^	NHANES 2013–2014			*p* Value
	FS ^1^	FI 1	FI 2		FS	FI 1	FI 2	
Depression ^3^								
Model 1 ^4^	1.00	2.3 (1.4–3.8) ^5^	5.1 (3.1–8.3)	<0.0001	1.00	2.3 (1.2–4.3)	3.8 (2.9–5.0)	<0.0001
Model 2	1.00	2.3 (1.4–3.8)	5.0 (3.1–8.2)	<0.0001	1.00	2.4 (1.2–4.7)	4.1 (3.2–5.3)	<0.0001
Model 3	1.00	1.9 (1.1–3.3)	4.0 (1.7–9.0)	<0.0001	1.00	2.0 (1.1–3.6)	3.0 (2.3–3.7)	<0.0001

^1^ The study subjects were divided into three groups according to their food insecurity status: (1) food secure group (FS), (2) mildly food insecure group (FI 1), (3) moderately-to-severely food insecure group (FI 2) ^2^ *p* value from multiple logistic regression analysis ^3^ Depression was defined as Patient Health Questionnaire-9 (PHQ-9) score < 10 normal, ≥10 depression ^4^ Model 1: Crude; Model 2: Adjusted for sex, age; Model 3: Adjusted for sex, age, income, marital status, education, chronic disease, physical disabilities, and menopausal status (only in women) in KNHANES; for sex, age, income, marital status, education, race/ethnicity, chronic disease, physical disabilities, and menopausal status (only in women) in NHANES; ^5^ OR (95% CI), odds ratios (ORs) from the mildly food insecure group (FI 1), moderately-to-severely food insecure group (FI 2) relative to the food secure group (FS). KNHANES: Korea National Health and Nutrition Examination Survey; NHANES: National Health and Nutrition Examination Survey.

## Data Availability

The datasets were analyzed in this study are available in publicly repository: http://knhanes.cdc.go.kr (KNHANES); https://wwwn.cdc.gov/nchs/nhanes/ContinuousNhanes/Default.aspx?BeginYear=2013 (NHANES).
